# Use of High Sensitivity GNSS Receiver Doppler Measurements for Indoor Pedestrian Dead Reckoning

**DOI:** 10.3390/s130404303

**Published:** 2013-03-28

**Authors:** Zhe He, Valérie Renaudin, Mark G. Petovello, Gérard Lachapelle

**Affiliations:** PLAN Group, Schulich School of Engineering, The University of Calgary, 2500 University Drive NW, Calgary, AB T2N 1N4, Canada; E-Mails: zhehe@ucalgary.ca (Z.H.); valerie.renaudin@ifsttar.fr (V.R.); mark.petovello@ucalgary.ca (M.G.P.)

**Keywords:** high sensitivity GNSS, indoor multipath, pedestrian dead reckoning, tight integration, Doppler measurements, direct vector processing

## Abstract

Dead-reckoning (DR) algorithms, which use self-contained inertial sensors combined with gait analysis, have proven to be effective for pedestrian navigation purposes. In such DR systems, the primary error is often due to accumulated heading drifts. By tightly integrating global navigation satellite system (GNSS) Doppler measurements with DR, such accumulated heading errors can usually be accurately compensated. Under weak signal conditions, high sensitivity GNSS (HSGNSS) receivers with block processing techniques are often used, however, the Doppler quality of such receivers is relatively poor due to multipath, fading and signal attenuation. This often limits the benefits of integrating HSGNSS Doppler with DR. This paper investigates the benefits of using Doppler measurements from a novel direct vector HSGNSS receiver with pedestrian dead-reckoning (PDR) for indoor navigation. An indoor signal and multipath model is introduced which explains how conventional HSGNSS Doppler measurements are affected by indoor multipath. Velocity and Doppler estimated by using direct vector receivers are introduced and discussed. Real experimental data is processed and analyzed to assess the veracity of proposed method. It is shown when integrating HSGNSS Doppler with PDR algorithm, the proposed direct vector method are more helpful than conventional block processing method for the indoor environments considered herein.

## 1. Introduction

A wide range of commercial applications such as emergency services and cell phone location-based services (LBS) have driven the development of pedestrian navigation technology over the past several years. With a demand for low cost and high reliability, attention has been given to using additional sensors or devices such as Wi-Fi, inertial sensors and ZigBee radios to integrate with GNSS receivers. In particular, inertial sensors that can provide DR information have proven to be of great potential [1–4]. For pedestrian navigation, the PDR algorithm is often utilized because it makes best use of the fact that users are most likely to move on foot, and the costs of the required inertial sensors are relatively low. Much research has thus been directed towards improving PDR algorithms, either on reliable step length detection or improved heading estimation, such as [5–8].

On the other hand, GNSS receivers are often used together with PDR algorithms due to the fact that their errors are not accumulated. Many researchers have investigated integrating PDR algorithms with global positioning system (GPS) receivers. The feasibility and performance of using a low-cost motion sensor integrated with GPS and differential GPS (DGPS) was assessed in [9]. The performance of using pedestrian dead-reckoning with a micro-electro-mechanical system (MEMS) inertial measurement unit (IMU) to aid high sensitivity GPS in harsh environment was reported in [10]. Generally speaking, the absolute accuracy of the integrated system is governed by the accuracy of the GNSS receivers.

In order to enhance the performance of the GNSS receiver, a typical method is to increase the coherent integration, as shown in [3]. However, extremely long coherent integration requires that stringent requirements be met, such as to compensate for user motion, which is not the focus of this paper. Instead, the spatial gains obtained by non-coherent integration among satellites are explored. As signal degradation is inherent for indoor GNSS signals, even if high sensitivity receivers are still able to generate measurements in such challenged environments, their quality is usually poor. For example, pseudorange observations in shopping malls or tower block buildings are largely biased and can result in 20 to 60 m horizontal root mean squared errors, even with commercial HSGNSS [11], and in these indoor environments, the benefits of integrating PDR with HSGPS are often limited due to multipath and fading. The performance of PDR integrated with conventional HSGPS using Doppler measurements in various indoor scenarios was assessed in [12]. Results showed that the performance improvement of integrating conventional HSGPS's Doppler measurements with PDR was bottlenecked by the quality of Doppler measurement. It also indicated that HSGPS Doppler which uses block processing techniques [13,14] in some indoor environments cannot provide beneficial Doppler measurements.

With this in mind, the major objective of this paper is to investigate a new method of generating HSGNSS Doppler measurements with the goal of improving PDR implementation in certain degraded signal scenarios. A direct vector processing method is thus proposed and developed. First, the velocity maximum likelihood estimate (MLE) is obtained. Then, Doppler measurements are generated based on such velocity MLE. The advantage of this approach is its reliability in harsh indoor environments where line of sight (LOS) and/or non-LOS (NLOS) signals are present. Subsequently, the benefit of these measurements for improving PDR algorithms indoors is investigated. The methodology proposed here is analyzed based on the indoor signal and multipath models, which are intrinsically related with the distribution of multipath statistics. Real experimental data is then presented to further verify the effectiveness of the proposed methodology.

The contributions of the paper are two-fold. First, a new direct vector processing receiver architecture is introduced and developed, which is shown to provide a more reliable velocity solution as well as Doppler measurements. Second, by using the new Doppler measurements integrated with PDR, the results are shown to improve the horizontal velocity accuracies by factors of more than 9% over the tradition implementation. Thus the effectiveness and benefits of the proposed Doppler estimation method are demonstrated and validated.

The paper is organized as follows: in Section 2, the signal and multipath models are introduced. After reviewing the architecture of conventional HSGNSS receivers, the proposed direct vector receiver is introduced. Then the velocity and Doppler estimation with direct vector processing in indoors are discussed in detail. In Section 3, the HSGNSS/PDR tight integration algorithm used in this paper is introduced. In Section 4, real indoor data is processed and analyzed. PDR-only solution, HSGPS/PDR tight integration with conventional Doppler and proposed Doppler measurements solutions are shown, compared, and discussed. Finally, conclusions are drawn in Section 5.

## 2. Direct Vector Processing in Indoor Multipath Environments

In this section, an indoor signal and multipath model is first introduced. The model is used to analyze how indoor multipath signals affect conventional HSGNSS Doppler estimation. After that, the proposed direct vector receiver architecture is introduced and discussed with comparison to the conventional HSGNSS receiver.

### 2.1. Signal and Multipath Model

The environment considered herein is indoors with dense multipath, where the multipath delay spread is usually smaller than one chip duration, or equivalently, the coherence bandwidth is much larger than the signal bandwidth (spreading code bandwidth in GNSS case). Under this scenario, a non-frequency selective channel or flat-fading channel is usually assumed which implies the multipath time-delay is non-resolvable [15].

Once the radio frequency signal is received by the antenna, the receiver down-converts it to near baseband. At this point, the general complex signal envelope can be expressed as:

(1) $$\begin{array}{cc} r_{l}(t)=\sum_{i=1}^{N_{\text{sat}}}a_{i}x_{i}(t-\tau_{i})h_{i}(t)+w(t), & N_{\text{sat}}\geq 1 \end{array}$$

In Equation (1) *N_sat_* is the number of satellites in view, *a_i_* is the signal amplitude, *τ_i_* is the line-of-sight (LOS) signal time delay, *h_i_*(*t*) is the channel gain series. *x*_i_(*t*) = *d*_i_(*t*)*p*_i_(*t*) is defined as the product of the spreading code or pseudo random noise (PRN) code, *p_i_*(*t*) and the navigation data bits, *d_i_*(*t*). *w*(*t*) is input additive white Gaussian noise. Here the noise *w*(*t*) is assumed independent of the signal and has a flat power spectrum over the pre-correlation bandwidth. In the indoor case, the channel gain series can be further broken down as follows [16]:

(2) $$\begin{array}{ll} h_{i}(t) & =\sqrt{\frac{K_{i}}{K_{i}+1}}e^{j(\omega_{Di,\text{max}}\cos\alpha_{0}\cos\varepsilon_{0}t+\phi_{0}^{\prime })}+\frac{1}{\sqrt{K_{i}+1}}\frac{1}{\sqrt{M_{i}}}\sum_{m_{i}=1}^{M_{i}}A_{m_{i}}(t)e^{j\omega_{D_{i},\text{Max}}\cos\alpha_{m_{i}}\cos\varepsilon_{m_{i}}t+j{\phi^{\prime }}_{m_{i}}}\\ & =h_{\text{LOS},i}(t)+h_{\text{NLOS},i}(t) \end{array}$$

From Equation (2), The NLOS channel gain has *M_i_* multipath components, *A_mi_, α_mi_, ε_mi_* are the weighting factor, azimuth and elevation angles for $m_{i}^{th}$ multipath component. *K_i_* is the Ricean factor for *i^th^* satellites, which is the ratio between LOS and NLOS signal powers. The subscript 0 represents LOS component, while subscript *m* represents one NLOS path. It is noted that the channel gain series is decomposed into two components; one for the LOS signals and one for all NLOS signals. The first term on the right hand side in the above equation is the LOS channel gain series. The term involving the summation is the channel gain due to NLOS signals. For convenience, the total channel gain series is defined to have unity power.

Having presented the basic signal model with dense multipath, attention is now given to how this signal is handled within a GNSS receiver, and how it affects the conventional HSGNSS Doppler estimation. The conventional block processing technique for Doppler measurement is discussed in [14] and is based on the Doppler frequency MLE. With the notation introduced above, the correlator output for *i_th_* satellite with code delay and Doppler frequency (*τ_I,j_*, *f_D,I,k_*) can be expressed as:

(3) $$y_{i}{\left[n\right]}_{\left(\tau_{i,j},f_{D,i,k}\right)}=r[n]x_{i}(nT_{s}-\tau_{i,j})e^{-j2\pi f_{D,i,k}nT_{s}}$$

where *x_i_*(*nT_s_*−*τ_i,j_*)*e*^−*j*2*πf*_*D,i,k*_*nT*_*s*_^ represents the local code multiplied with the local carrier replica, which has a code delay of *τ_i,j_* and a Doppler frequency shift of *f_D,i,k_* where *k* ∈ [1, 2, …, *K*] represents the indices of the searching range for Doppler frequency.

If the coherent integration interval uses *N* samples, the Doppler MLE of a single satellite can be obtained from Equation (3) as follows:

(4) $$\begin{array}{cc} {\hat{f}}_{D,i} & =\arg\underset{k=1:K}{\max}\frac{1}{N}{\left|\sum_{n=0}^{N-1}y_{i}{\left[n\right]}_{\left(\tau_{i,j},f_{D,i,k}\right)}\right|}_{\tau_{i,j}=\tau_{i,0}}^{2}\\ & =\arg\underset{k=1:K}{\max}\frac{1}{N}{\left|\sum_{n=0}^{N-1}r[n]x_{i}(nT_{s}-\tau_{i,j})e^{-j2\pi f_{D,i,k}nT_{s}}\right|}_{\tau_{i,j}=\tau_{0}}^{2}\\ & \approx \arg\underset{k=1:K}{\max}\frac{a_{i}^{2}}{N}{\left|\sum_{n=0}^{N-1}h_{i}[n]e^{-j2\pi f_{D,i,k}nT_{s}}\right|}^{2}\\ & =\arg\underset{k=1:K}{\max}a_{i}^{2}I_{i}(f_{D,i,k})\approx \arg\underset{k=1:K}{\max a_{i}^{2}P_{i}(f_{D,i,k})} \end{array}$$

In Equation (4), it is apparent that the single Doppler frequency estimation will be directly affected by the channel gain series statistics. More precisely, this test statistic is *I_i_*(*f*), which denotes periodogram of the channel gain series. This statistic is an asymptotically unbiased spectral estimator [17] of the true power spectral density (PSD) of channel gain series *i.e.*, *P_i_*(*f*). Due to the fact that all estimators are based on time-limited sequences, the actual spectrum is the PSD of channel gain series convolved with spectrum of the time window. However, the periodogram is a reasonable approximation of actual PSD. So throughout the paper, the ideal PSD of channel gain series (*P_i_*(*f*) for *i^th^* satellite) is investigated instead of *I_i_*(*f*).

In the vector form, the Doppler MLEs for all available satellites will be:

(5) $${\hat{\text{f}}}_{D}=\arg\underset{k=1:K}{\max}\sum_{i=1}^{N_{\text{sat}}}a_{i}^{2}I_{i}(f_{D,i,k})\approx \arg\underset{k=1:K}{\max}\sum_{i=1}^{N_{\text{sat}}}a_{i}^{2}P_{i}(f_{D,i,k})$$

From Equation (4) and (5), it is also observed that for individual Doppler MLE, each channel or satellite is processed independently.

When a channel is time varying, the so called “Doppler spread” is sometimes introduced [18]. This term is defined as frequency spread in the signal spectrum due to different signal propagation paths contributing to a single fading channel. Consequently, in some non-symmetric ring scattering environments (*i.e.*, where multipath from certain directions is much stronger than other direction), the NLOS components dominate the signal. Then the PSD of the channel gain series will be significantly distorted by certain channel parameters, and Doppler MLE can be biased. The most important channel parameters in this regard are the multipath angle-of-arrivals (AOAs). As such, once the distribution of the multipath AOA is specified, the ideal PSD of the channel gain can be derived [16]. In a more compact form, if the NLOS carrier power spectrum is expressed as a function of multipath AOAs, then the overall carrier power spectrum has the following form:

(6) $$\begin{array}{cc} P_{i}(f) & =\frac{1}{K_{i}+1}P_{\text{NLOS},i}(f)+\frac{K_{i}}{K_{i}+1}P_{\text{LOS},i}(f)\\ & =\frac{1}{K_{i}+1}g_{\text{NLOS},i}(f,\alpha_{\text{avg},i},\beta_{\alpha ,i},\varepsilon_{\text{avg},i},\beta_{\varepsilon ,i})+\frac{K_{i}}{K_{i}+1}\delta (f-f_{D,i}) \end{array}$$

In Equation (6), *K_i_* is the Ricean factor, *f_D,i_* is the LOS Doppler, *α_avg,i_*, *ε_avg,i_*, *β_α,i_, β_ε,i_* respectively represent averaged multipath azimuth, elevation angle and beamwidth for azimuth and elevation angles. Function *g_NLOS,i_* describes the major power distribution of all multipath components for the *i^th^* satellite. Its shape and centre are determined by the above mentioned multipath AOA parameters. In Equation (6), when the Ricean factor *K* is extremely small, multipath will dominate the signal and the Doppler MLE for this satellite may be biased. It is noted that as coherent integration time is extended, the LOS signal power might be accumulated to the point where it is usable, and the multipath power might decrease due to exceeding the coherence time of fading channel. However it is not practical to unlimitedly increase the coherent integration time, which usually calls for stringent requirements for both oscillator and inertial systems. In real scenarios, it is more common to have combined LOS and NLOS signals or NLOS-only signals present in the correlator outputs, which are the focus of the paper.

### 2.2. Conventional and Direct Vector GNSS Software Receiver Architectures

**Figure 1.**
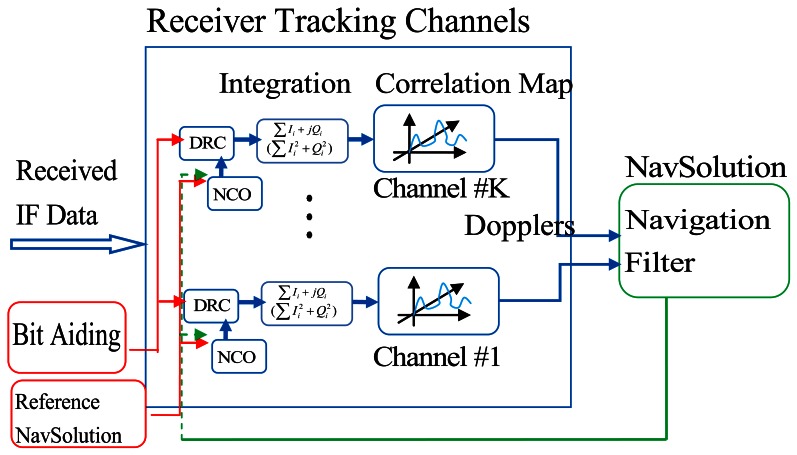
Conventional high sensitivity GNSS receiver architecture.

The architecture of a conventional high sensitivity GNSS software receiver that uses block processing technique is illustrated in Figure 1. The received intermediate frequency (IF) data is first fed to a Doppler removal and correlation (DRC) block. After the integration module, which might have coherent or non-coherent form, the despread and demodulated signals are then processed by a block processing technique. The pseudorange and Doppler measurements are estimated by selecting the maximum power in the correlator outputs (code phase and Doppler domain). With such measurements, the user position and velocity can be estimated by either least squares or Kalman filtering. When it comes to weak signal conditions, these measurements are often themselves biased and lead to very large residuals. An effective way to deal with such measurements is to weight them according to their accuracies. Due to the fact that the Doppler estimation accuracy is inversely proportional to the received signal power, once accurate signal power can be estimated, such weighting can be applied. However, the C/N_0_ estimation will get worse under weak signal conditions, which limits the benefits of using conventional HSGNSS Doppler measurements.

**Figure 2.**
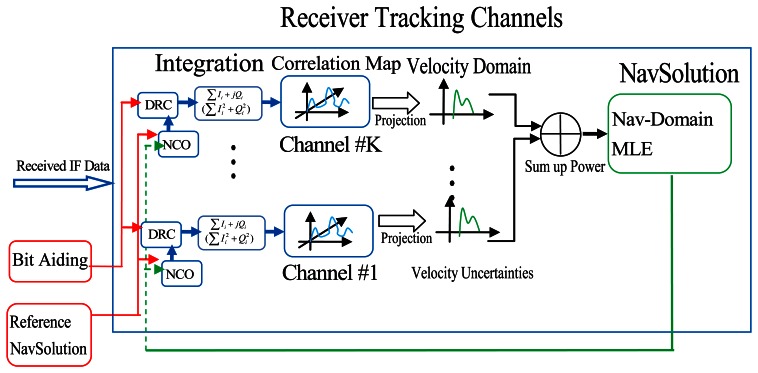
Direct vector GNSS receiver architecture.

Direct vector receiver architecture is thus proposed in Figure 2. With scrutiny of the figure, one can observe that in direct vector receiver, the signal power in the Doppler domain for all satellites is projected onto the velocity domains. In this way, the estimated velocity solutions are based on all the information contained in all correlator outputs across all satellites which is essentially the velocity MLE.

As for conventional high sensitivity GNSS receivers, the velocity estimation is only based on the Doppler MLEs, which discards some information before final solution is made. Another major benefit of the direct vector receiver is that weighting is automatically performed according to the received signal strength [19]. In such direct vector processing, all the power in correlator domain is preserved until a navigation solution is obtained. For example in this paper, once a valid velocity solution is obtained, the Doppler measurement for each satellite can be generated accordingly. Details of how the velocity MLE in the presence of multipath signals is obtained are discussed in the following section.

### 2.3. Direct Vector Processing in Indoor Multipath

It is known that the measured Doppler frequency from GNSS receivers has the following relationship with the user velocity:

(7) $$\begin{array}{cc} f_{D,i}(\text{v})=\frac{1}{\lambda }(u_{i,x}(\nu_{u,x}‐\nu_{\text{sat},i,x})+u_{i,y}(\nu_{u,y}‐\nu_{\text{sat},i,y})+\cdots u_{i,z}(\nu_{u,z}‐\nu_{\text{sat},i,z}))+\frac{1}{\lambda }c\dot{d}t, & i=1,2...,N_{\text{sat}} \end{array}$$

where λ is the wavelength of transmitted RF signal, *ν_u,x_, ν_u,y_,ν_u,z_* are the user ECEF user velocities, *i* is the index of current satellite, *ν_sat,i,x_*, *ν_sat,i,y_*, *ν_sat,i,z_* are the ECEF satellite velocities, **u***_i_* = [*u_i,x_*, *u_i,y_*, *u_i,z_*] is the direction unit vector from the satellite to the receiver, *cdṫ* is the receiver clock drift. As such, the user velocity vector is assumed to also include the clock drift term, *i.e.*, **v***_u,xyz_* = [*ν_u,x_*, *ν_u,y_*, *ν_u,z,_ cd˙t*]. Due to the invariance property of maximum likelihood estimates [20], the velocity MLE can then be easily obtained by:

(8) $${\hat{\text{v}}}_{ML}=\arg\underset{\text{v}}{\max}\left\{\sum_{i=1}^{N_{\text{sat}}}a_{i}^{2}I_{i}(\text{v})\right\}\approx \arg\underset{\text{v}}{\max}\left\{\sum_{i=1}^{N_{\text{sat}}}a_{i}^{2}P_{i}(\text{v})\right\}.$$

From Equation (8), one can see that the final velocity solution is obtained by weighting available carrier power spectrums for all satellites. The weighting factors are automatically chosen as the actual signal powers (*a_i_*^2^). The physical interpretation of velocity MLE can be considered as follows. First, the LOS signal power which is an impulse in the Doppler domain can be projected to the velocity domain on a satellite by satellite basis. Analogously, the NLOS signal power can also be projected into the velocity domain. The point in the velocity domain that has the greatest power will represent the final estimate. In the following, how the velocity MLE is obtained in the presence of indoor multipath signals will be shown in detail.

In order to project the signal power from the Doppler domain to the velocity domain, the relationship between the Doppler to the velocity can be used. For example, a small offset in the Doppler will cause a small offset in the velocity, these two terms are linearly related as shown in Equation (9):

(9) $$\Delta f_{D,i}=\left[\frac{1}{\lambda }u_{i,x},\frac{1}{\lambda }u_{i,y},\frac{1}{\lambda }u_{i,z},\frac{1}{\lambda }\right]\Delta {\text{v}}_{u,\text{xyz}}={\text{a}}_{i}^{T}\Delta {\text{v}}_{u,\text{xyz}}={\text{a}}_{i}^{T}C_{n}^{e}\Delta {\text{v}}_{u,\text{enu}}={\text{e}}_{i}^{T}\Delta {\text{v}}_{u,\text{enu}}$$

In Equation (9), *e_i_^T^* is the projection vector from velocity to the Doppler for *i^th^* satellite. *C_n_^e^* is the rotation matrix from navigation frame to the earth frame. Δ**v***_u,enu_* is the user velocity offset vector with respect to the velocity searching centre in navigation frame which also includes the clock drift term. Δ*f_D,i_* is the Doppler frequency offset for this satellite relative to Doppler searching centre.

**Figure 3.**
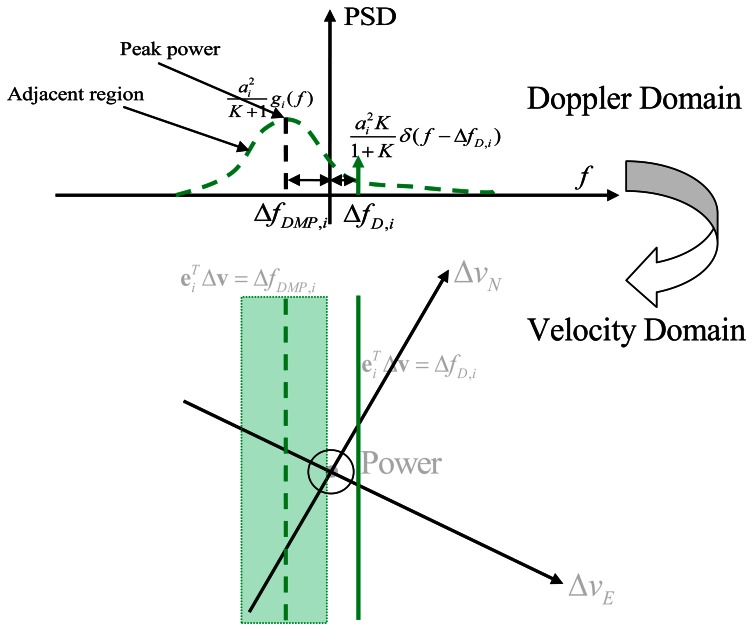
Power projection from Doppler domain to velocity domain (2D case).

In order to visualize the effect of multipath on velocity estimation, it is convenient to first consider the two dimensional case. Assuming vertical velocity and clock drift are already known or constrained, the Doppler offset is then only related with two horizontal velocity offsets (Δ*ν_E_* and Δ *ν_N_*). The carrier PSD over the Doppler domain is shown in the upper part of Figure 3. This is based on Equation (6). The solid green line is the LOS signal power given by $\frac{a_{i}^{2}K_{i}}{K_{i}+1}$ and is located at the LOS Doppler offset,. Δ*f_D,i_*. On the other hand, the major NLOS signal power is distributed over a small region, and has a maximum power of $\frac{a_{i}^{2}}{K_{i}+1}g_{i}(\Delta f_{\text{DMP},i})$ with a frequency offset of Δ*f_DMP,i_*. In the velocity domain, the LOS signal is equally distributed over the line or plane ${\text{e}}_{i}^{T}\Delta {\text{v}}_{u}=\Delta f_{D,i}$. Similarly, for the NLOS power, the peak power is along the line or plane ${\text{e}}_{i}^{T}\Delta {\text{v}}_{u}=\Delta f_{\text{DMP},i}$, and powers of its adjacent region can be evaluated with different offsets according to Equation (6).

**Figure 4.**
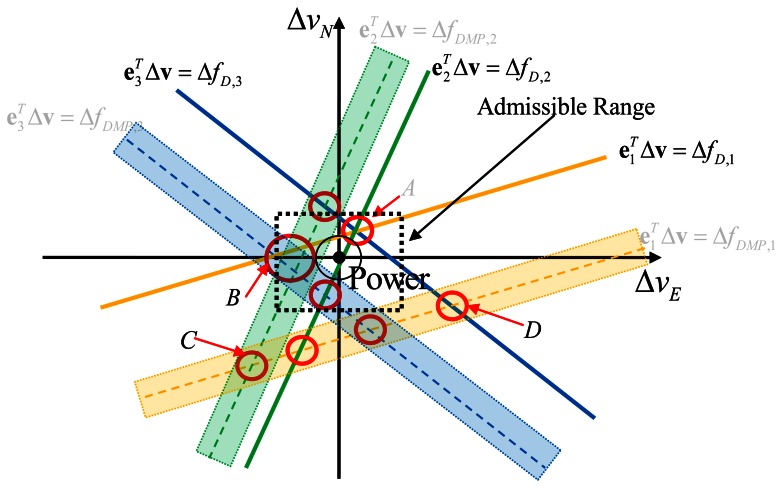
Velocity MLE with channel distortion (2D case).

As more satellites are considered, the situation tends to that shown in Figure 4. In this case, there are three satellites considered. All three LOS powers intersect at the point *A*, so the power of point *A* denotes the total power for LOS signals. It is also shown that there are other intersection points, such as point *B* (two NLOS power together with one LOS), point *C* (two NLOS power only), and point *C* (one NLOS power and one LOS power). The more intersections located at a particular point, the more power will be observed, and the more probable it will be the final velocity estimate. In the velocity domain, the point that contains the maximum power will be considered as the final estimate of the velocity.

The velocity estimation procedure shown in Figure 4 relies on many factors. Under different scenarios, it may have totally different performance. Firstly, if all LOS components are dominating the signal, or equivalently, the Ricean factors for all three satellites are very large, then point *A* will naturally be the final estimate and should be similar to the least squares velocity estimation that processes Doppler MLEs independently. Secondly, if only one LOS component has a large Ricean factor, say the yellow curve, and the other two satellite signals have dominant NLOS components, then the power of point *A* may have less power than that of point *B* thus potentially making point *B* the final estimate. In this case, the result is not based on LOS signals only, but with partial LOS information and partial NLOS information. In other scenarios, it is equally possible that point *C* or point *D* might have the maximum power, which should be avoided. Consequently an admissible region is defined in order to discard such outliers, and the size of the regains varies according to different user activities. For example, the horizontal acceleration during walking in this paper is assumed within the range ±2 *m*/*s*^2^.

On the other hand, as the number of satellites tracked increases, the LOS signal power in the velocity domain is accumulated without loss, while the NLOS signal power in velocity domain is usually dispersed. The reason is that the projection matrix $\text{E}=[{\text{e}}_{1}^{T};{\text{e}}_{1}^{T};{\text{e}}_{3}^{T}$ from user velocity to LOS Doppler offset array (Δ**f***_D_*) is calculated for LOS signal propagation. In other words, Δ**f***_D_* is in the column space of the projection matrix. For the NLOS Doppler offset array, this is generally not true. The other reason is that the multipath statistics for each satellite are generally different and time varying, whereas the LOS signal is predictable and deterministic. If by accumulating power between satellites, the total power of LOS components continues to increase and if it surpasses all the dispersed NLOS power, a relatively good velocity estimate will occur. Another acceptable scenario is similar to point *B* in the Figure 4, where the power is highest with a combination of LOS and NLOS components. If more than two LOS powers intersect at point *B* along with other NLOS power, the final estimate then takes advantages of both LOS and NLOS signal components.

The above section discusses the power projection in two dimensional spaces. In the following, the same principles are extended to the real scenario where four velocity states need to be estimated. Assuming there are N*_sat_* available satellites, the four-dimensional hyper-planes of LOS powers for all satellites are given by:

(10) $$\{\begin{array}{c} {\text{e}}_{1}^{T}\Delta {\text{v}}_{u,\text{enu}}=\Delta f_{D,1}\\ {\text{e}}_{2}^{T}\Delta {\text{v}}_{u,\text{enu}}=\Delta f_{D,2}\\ \vdots \\ {\text{e}}_{N_{\text{sat}}}^{T}\Delta {\text{v}}_{u,\text{enu}}=\Delta f_{D,N_{\text{sat}}} \end{array}\text{or}\;\text{E}\Delta {\text{v}}_{u,\text{enu}}=\Delta {\text{f}}_{D}$$

Each hyper-plane is associated with the LOS signal power of $\frac{a_{1}^{2}K_{1}}{1+K_{1}},\frac{a_{2}^{2}K_{2}}{1+K_{2}},\ldots ,\frac{a_{N_{\text{sat}}}^{2}K_{N_{\text{sat}}}}{1+K_{N_{\text{sat}}}}$. Analogously, the NLOS signal power is distributed over the hyper-planes and has a certain amount of power according to *g_NLOS,i_* in Equation (6) with different frequency offsets:

(11) $$\{\begin{array}{c} {\text{e}}_{1}^{T}\Delta {\text{v}}_{u,\text{enu}}=\Delta f_{\text{DMP},1}\\ {\text{e}}_{2}^{T}\Delta {\text{v}}_{u,\text{enu}}=\Delta f_{\text{DMP},2}\\ \vdots \\ {\text{e}}_{N_{\text{sat}}}^{T}\Delta {\text{v}}_{u,\text{enu}}=\Delta f_{\text{DMP},N_{\text{sat}}} \end{array}\text{or}\;\text{E}\Delta {\text{v}}_{u,\text{enu}}=\Delta {\text{f}}_{\text{DMP}}$$

For example, the peak powers associated with each hyper-plane of NLOS signal are $\frac{a_{1}^{2}}{1+K_{1}}g_{1}(\Delta f_{\text{DMP},1}),\frac{a_{2}^{2}}{1+K_{2}}g_{2}(\Delta f_{\text{DMP},2}),...,\frac{a_{N_{\text{sat}}}^{2}}{1+K_{N_{\text{sat}}}}g_{M}(\Delta f_{\text{DMP},N_{\text{sat}}})$. All hyper-planes described in Equations (10) and (11) finally form the velocity domain. The point in velocity domain associated with the maximum power will be considered as the velocity MLE.

The velocity MLE can be obtained by using the Equations (8), (9), (10), and (11). The offset associated with maximum power in the velocity domain is considered as final estimate of velocity. Once the velocity MLE is obtained, the Doppler measurements for each satellite can then be computed according to Equation (9). In this direct vector processing method, the Doppler measurements are based on maximum use of LOS/NLOS signal power and maximum use of mutual information between each satellite. In this way, a certain amount of information shared among satellites (see Figure 4) is fully utilized before arriving at a result. In contrast, conventional block processing methods only use Doppler MLE, whose residual can be quite large in indoor environments, potentially causing the solution not to converge. In the following sections, the Doppler estimated from block processing and direct vector processing methods will be evaluated and compared by integrating with PDR algorithms with real experimental data.

## 3. HSGPS/PDR Tight Integration

In this section, the system model for the HSGNSS/PDR tight integration is first introduced. The measurement or observation models are then presented. Following this, the integration performance of using conventional and the proposed Doppler measurements with a PDR algorithm will be assessed in the next section.

### 3.1. System Model

The system state vector for the PDR filter is shown in Equation (12) and includes (in order) the user's 3D coordinates in the local level frame, the horizontal and vertical speeds, the walking heading direction and the GNSS receiver's clock drift in units of range rate (*i.e.*, multiplied by the speed of light, *c*):

(12) $$\text{x}=\left[E,\;N,\;U,\;v_{h},\;\theta ,\;v_{v},\;c\dot{d}t\right]$$

The GNSS receiver clock bias is not present, since in this paper only the Doppler measurements are used to update the integration filter. In turn, this is because the paper focuses on assessing the benefit of the proposed Doppler measurements for PDR.

The system dynamic model of the pedestrian's position follows the equations of a classical PDR mechanization and is given by:

(13) $$\begin{array}{l} \dot{E}=v_{h}\sin(\theta )\\ \dot{N}=v_{h}\cos(\theta )\\ \dot{U}=v_{v} \end{array}$$

The velocities and the heading are further modeled as random walk processes:

(14) $$\begin{array}{l} {\dot{v}}_{h}=\eta_{v_{h}}\\ {\dot{v}}_{v}=\eta_{v_{v}}\\ \dot{\theta }=\eta_{\theta } \end{array}$$

Similarly, the clock drift state is modeled as:

(15) $$c\ddot{d}t=\eta_{\text{cdt}}$$

In Equations (14) and (15), *η_νh_*, *η_νu_*, *η_θ_* and *η_cdt_* are the white Gaussian driving noise of the corresponding state elements. The spectral density of the clock drift error noise is computed using a standard clock stability model, as found in [21].

It is noted that discrepancies between PDR-derived velocity and GNSS-derived velocity are expected. In particular, due to the repetitive nature of the human gait, Doppler measurements typically exhibit oscillations over the course of a full gait cycle. In contrast, by its very nature, PDR velocities are effectively averaged over the course of a step and do not contain these oscillations, thus introducing an oscillatory discrepancy between the PDR- and GNSS-derived values. Correspondingly, these oscillations should be modeled to properly integrate the velocity information based on Doppler measurements.

For coping with these oscillations over a step, the classical PDR has been modified in this work. Normally, the filter state is only propagated when a step is detected. In the proposed approach, however, Doppler measurements are used at each GNSS measurement epoch, typically at a higher rate than the step frequency. As such, the PDR filter is propagated at this higher rate as well. However, PDR observations are still only used when a step is detected. It is expected that this approach will average the oscillating effects sensed by Doppler measurements over one step. The advantage of this asynchronous measurement update is that the integrated system could track the rapid changes of the heading sensed by the IMU or other heading sensors.

On the other hand, there are also some disadvantages with this new integration method. First, the computational load is increased because the filter is propagated more frequently. In this paper, a coherent integration time of 500 ms is used, such that the measurement update time for Doppler is 2 Hz, so computational load is not a major concern. Second, as the coherent integration time increases, the Doppler measurement actually conveys information about the average velocity (and thus attitude) during the integration interval. However, this type of averaged Doppler measurement is still usable since it can help to alleviate the long term heading drift of the PDR system.

### 3.2. Measurement Model

Having introduced the system model of the integration filter, the following discusses the measurement models. There are two types of measurement updates for the proposed tight integration; from the PDR and from the GNSS Doppler.

The PDR sensor update is composed of step length updated and heading update. The measured step length and walking directions are related to the user's position and velocity through the following equations:

(16) $$\begin{array}{l} s=v_{h}\Delta t_{\text{step}}+\eta_{SL}\\ \theta_{\text{obs}}=\theta +\eta_{\theta } \end{array}$$

where *s* is the user's step length, Δ*t_step_* is the step duration and *θ_step_* is the averaged heading over the last step.

The measured GNSS Doppler is related to the pedestrian's velocity via Equations (7) and (9). In order to be consistent with other observation models, it is listed again here:

(17) $$f_{D,i}(\text{v})=\frac{1}{\lambda }(u_{i,x}(v_{u,x}‐v_{\text{sat},i,x})+u_{i,y}(v_{u,y}‐v_{\text{sat},i,y})+\cdots u_{i,z}(v_{u,z}‐v_{\text{sat},i,z}))+\frac{1}{\lambda }c\dot{d}t+\eta_{f}$$

where *η_f_* is the noise induced by the receiver. After linearization of Equation (17), then the observation equation is:

(18) $$\begin{array}{ll} \delta {\text{f}}_{D} & ={\left[\begin{array}{c} {\hat{f}}_{D,1}\\ {\hat{f}}_{D,2}\\ \vdots \\ {\hat{f}}_{D,N_{\text{sat}}} \end{array}\right]}_{\text{Filter}}-{\left[\begin{array}{c} f_{D,1}\\ f_{D,2}\\ \vdots \\ f_{D,N_{\text{sat}}} \end{array}\right]}_{Rx}=\frac{1}{\lambda }{\text{H}}_{\dot{\rho },M\times 3}\left[\begin{array}{c} \delta v_{e}\\ \delta v_{n}\\ \delta v_{u} \end{array}\right]+\frac{1}{\lambda }1_{M\times 1}\delta \text{cdt}+\left[\begin{array}{c} \eta_{f_{D},1}\\ \eta_{f_{D},2}\\ \vdots \\ \eta_{f_{D},N_{\text{sat}}} \end{array}\right]\\ & \;=\left[\begin{array}{cc} \frac{1}{\lambda }{\text{H}}_{\dot{\rho },N_{\text{sat}}\times 3}T_{3\times 3} & \frac{1}{\lambda }1_{N_{\text{sat}}\times 1} \end{array}\right]\;\left[\begin{array}{c} \delta v_{h}\\ \delta \theta \\ \delta v_{v}\\ \delta \text{cdt} \end{array}\right]+\eta_{f} \end{array}$$

In Equation (18), ${\left[\begin{array}{cccc} {\hat{f}}_{D,1} & {\hat{f}}_{D,2} & \cdots & {\hat{f}}_{D,M} \end{array}\right]}_{\text{Filter}}^{T}$ are the filter predictions and ${\left[\begin{array}{cccc} f_{D,1} & f_{D,2} & \cdots & f_{D,M} \end{array}\right]}_{Rx}^{T}$ are the measurements from the receiver. The subscript *N_sat_* before *N_sat_* was used denotes the number of satellites. $H_{\dot{\rho },1\times 3}=\left[-\cos(\varepsilon )\sin(\alpha ),\;-\cos(\varepsilon )\cos(\alpha ),\;-\sin(\varepsilon )\right]$ is the design matrix from velocities to pseudorange rates, *ε* and *α* are the elevation angles and azimuth angles for each satellite. *λ* is the wavelength of the transmitted GNSS signal, and matrix *T*_3×3_ is defined as:

(19) $$T_{3\times 3}=\left[\begin{array}{ccc} \sin(\theta ) & v_{h}\cos(\theta ) & 0\\ \cos(\theta ) & -v_{h}\sin(\theta ) & 0\\ 0 & 0 & 1 \end{array}\right]$$

It is noted that PDR only provides information in the horizontal plane rendering the vertical velocity unobservable except with Doppler observations. To further constrain the vertical component of the pedestrian's position, barometer records could be used, although this was not done here. Similarly, indoors, pedestrians are mainly walking on flat surfaces and only change their elevation when climbing/descending stairs or when taking an elevator.

## 4. Data Processing and Analysis

This section deals with real experimental data processing and analysis in order to assess the benefits of integrating conventional and proposed HSGNSS Doppler measurements with PDR sensors. First, the data collection is described briefly. Then the analysis and results are described. For simplicity, only GPS satellites are used, but it is expected that results would likely improve if a multiple GNSS constellation were used.

### 4.1. Data Collection Description

**Figure 5.**
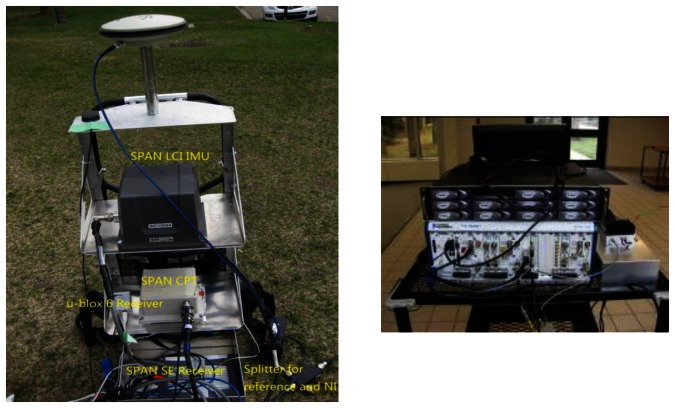
Primary equipments used in data collection. SPAN™ LCI IMU, CPT, and SPAN™ receivers are shown on the left; NI front-end is shown on the right.

The experimental data was collected on the campus of the University of Calgary. The primary pieces of equipment were an NI front-end, a Novatel SPAN receiver and a LCI IMU [22], all of which are shown in Figure 5, mounted on a backpack.

**Figure 6.**
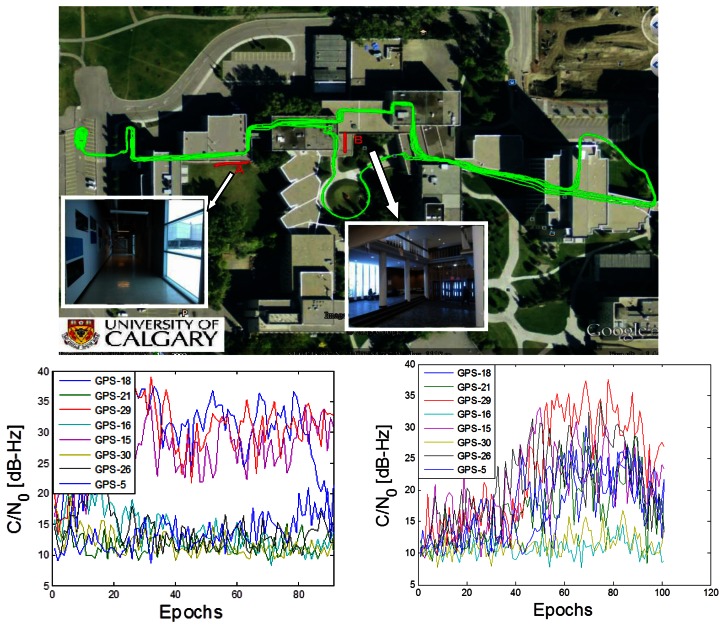
Data collection actual trajectory, indoor scenarios, and corresponding C/N0 plots (one epoch is 0.5 s)—scenario A picture taken facing east, scenario B picture taken facing south.

The raw IF data was collected with the NI front-end at a rate of 5 Msps (complex). The reference trajectory is shown in Figure 6. This trajectory is generated using Novatel Inertial Explorer™ with a Novatel SPAN™ system. The SPAN™ system used here includes a tactical grade IMU (LCI) and a high precision GNSS receiver, *i.e.*, SPAN™ receiver. The Inertial Explorer™ uses differential GNSS measurements to tightly integrate with IMU. However, the high precision SPAN™ receiver cannot provide GNSS measurements indoors due to the fact that the signal C/N_0_ is too weak for standard tracking loops. In such cases, the solution is computed only using IMU measurements. In order to get better results, both forward/backward smoothing and coordinate updates are used. By using this approach, the estimated position standard deviation was better than 3 m at all times, and the estimated velocity standard deviation was better than 0.02 m/s.

The trajectory contains several different indoor environments, and two spots are chosen to emphasize how Doppler from direct vector HSGPS outperforms that from conventional HSGPS (scenario A and scenario B). The pedestrian carries a backpack containing an antenna, the NovAtel SPAN system and LCI IMU. The cable from the backpack is connected to the NI front-end.

One of the benefits of the proposed direct vector processing is its autonomous weighting by power (equivalent to C/N_0_). Ideally, if it is possible to get accurate enough C/N_0_ estimates in the conventional HSGPS, the results can be very close to the proposed approach. However, there are some difficulties for C/N_0_ estimation in weak signal and multipath conditions. As shown in Figure 6, C/N_0_ values are fluctuating during the indoor periods. This is caused by the user-satellite dynamic and multipath (or fading) phenomenon. The other fact is that, as the signal is weak along with multipath, the C/N_0_ estimator will itself exhibit a larger variance or even be biased. By using these erroneous C/N_0_ weights, the conventional HSGPS may perform even worse.

**Figure 7.**
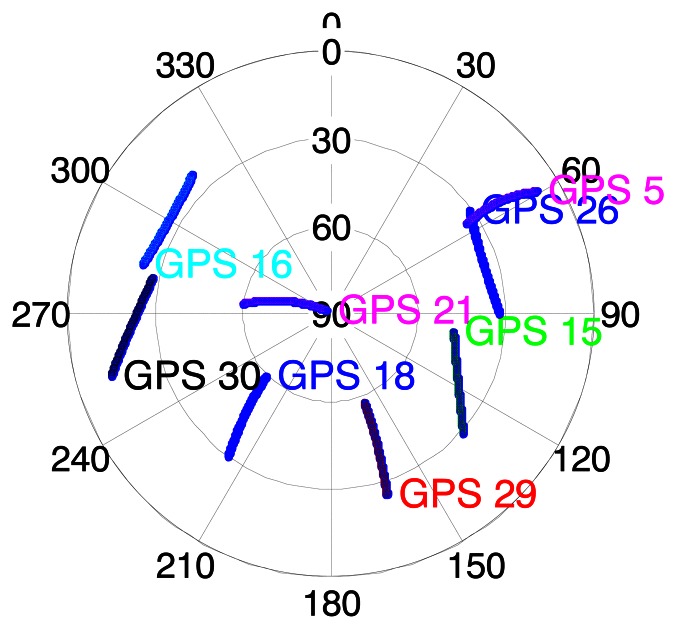
Sky plots of all available GPS satellites.

The sky plot of all available GPS satellites is shown in Figure 7.

To begin the test, the pedestrian walks in a circular path outside the building in order to align the inertial system. Then, the user walks through the inside of the building with periodic returns outside in order to maintain an accurate reference solution (details below). Finally, circular motion is repeated again at the end of the test in open sky scenario in order to facilitate backward processing of the data.

The raw IF data was processed using the GNSRx-ss™ software receiver [23] in order to get the raw correlator values over a pre-defined Doppler search range from which Doppler MLE can be easily obtained. In the GSNRx-ss™ receiver, assistance information such as raw data bits, nominal trajectory and broadcast ephemerides are also provided as input. At each measurement epoch, the reference trajectory is used to compute nominal pseudorange and Doppler values for each satellite in view, which are then passed to the signal processing channels. Each channel then computes a grid of correlators around the nominal code phase and Doppler values. Finally, the maximum correlator outputs are used to generate pseudorange and Doppler measurements. In practice, a reference solution would not be available to control the center of the search range. The major impact of this is that the search range in the Doppler domain would likely have to increase in order to accommodate the resulting velocity errors and resulting in an increased computation load. As long as the searching space increases, the same estimation performance has been demonstrated.

GSNRx-ss™ with a coherent integration of 500 ms is used. With this coherent integration time, it is expected that a desirable pre-detection SNR can be obtained. After projecting the correlator outputs onto the velocity domain, the velocity powers are computed using Equations (8) and (9).

In Figure 6, the C/N_0_ profiles of abovementioned two indoor scenarios are also shown. In scenario A, one can see that the power of three satellites is relatively stronger than the others, being in the range of 20 to 37 dB-Hz. The other five satellites have very weak signals in the range of 10 to 15 dB-Hz. Given the sky plot shown in Figure 7 and the picture of scenario A in Figure 6, one can assume that the satellite in the south part of the sky can penetrate the south-facing windows, resulting in larger C/N_0_ values, such as PRN 15, 18 and 29. In scenario B, one can see that C/N_0_ values of six satellites increase to between 15 and 35 dB-Hz during epoch 40 to 100. Referring to the sky plot in Figure 7 and the picture in Figure 6 one can see that the signals transmitted by the satellites from the south and east portions of the sky penetrate the windows more easily, such as PRN 29 PRN 15.

### 4.2. Velocity MLE

**Figure 8.**
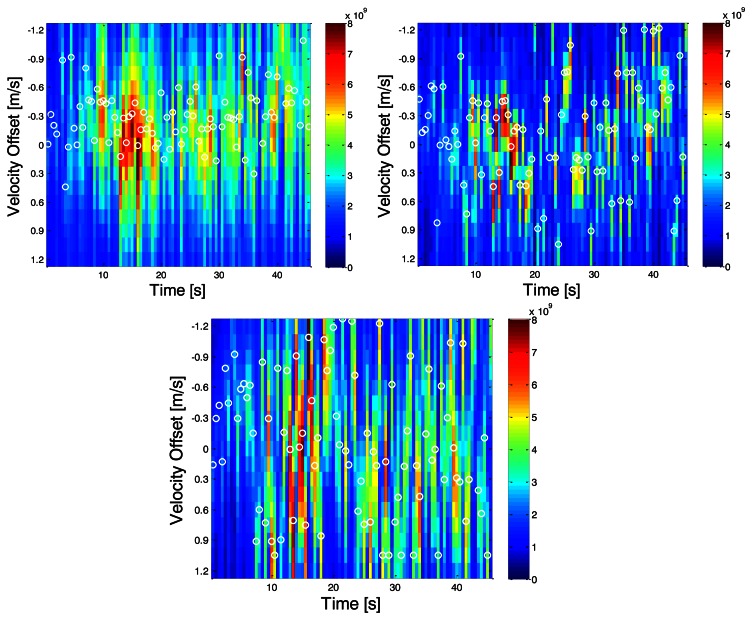
Power distributions in east, north and up velocity domains (**top left**, **top right** and **bottom**)–Scenario A.

**Figure 9.**
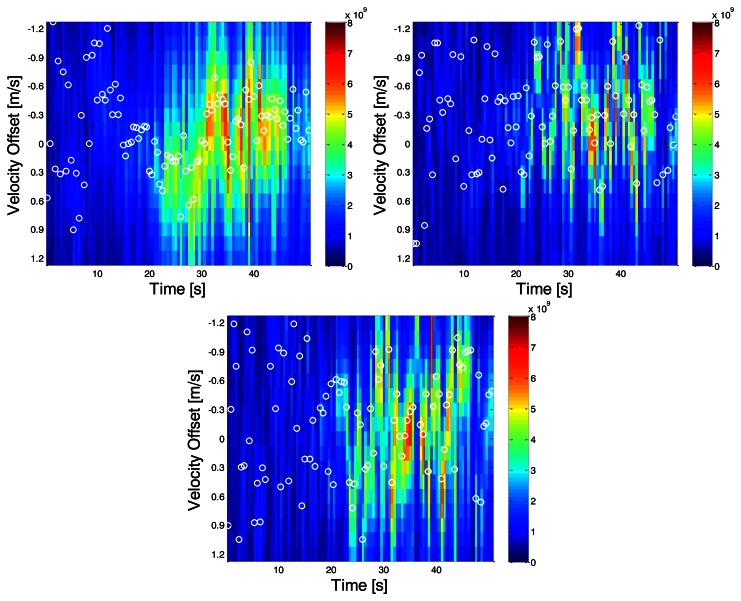
Power distributions in east, north and up velocity domains (**top left**, **top right** and **bottom**)–Scenario B.

Once correlator outputs from the receivers are available, the MLE velocities are computed first. The resulting velocity domain power distributions in the two indoor environments are depicted in Figures 8 and 9. Vertical axes are the velocity offsets with respect to the reference solution computed by using the NovAtel SPAN™ system. Horizontal axes are the time axes. The white circles represent the maximum powers at a given epoch. And the color of any one pixel in the image represents instantaneous received signal power across all satellites in that candidate velocity solution. Due to the fact that the centre of the bins is aligned with the user's true velocity, if the LOS signals are dominant, the largest power should be located at the centre.

In scenario A, it can be observed that around epoch 15 s, the total received power is the greatest and the dominant power is located very near the reference solution; the LOS signal appears to dominate the signal. However in the succeeding epochs, NLOS signals seem to dominate the received signal. The error statistics of the proposed direct vector and conventional HSGPS velocity solutions for this scenario are summarized in Table 1. One can see that the proposed algorithm outperforms the conventional one by 75%, 75%, and 89% in the east, north and up velocity axis, respectively.

Since the multipath statistics are environment-dependent, it is useful to show that the proposed algorithm works in various indoor environments. In Figure 9, the distribution of the total received signal power over velocity domains is plotted. It can be observed that the power is concentrated and consistent from time 20 to 40 s. Along with C/N0 plots, it is likely that the subject is approaching the windows during this period. The RMS velocity errors of the proposed direct vector and conventional HSGPS are summarized in Table 2. One can see the proposed algorithm outperforms the conventional one by 24%, 72%, and 83% in the east, north and up velocity axis, respectively.

Comparing results of the above two scenarios, two major phenomena are observed. First, the accumulated power fluctuates more from epoch to epoch while indoors. Second, the location of the peak power in the velocity domain largely depends on the multipath statistics or environments.

### 4.3. HSGPS/PDR Integration Performance

In direct vector processing, the velocity MLE is first obtained, and then the corresponding Doppler measurements are computed. It is then convenient to assess the performance of the HSGPS/PDR tight integration using various types of observation. In the following paragraphs, the performance of the PDR only navigation solution and HSGPS/PDR tight integration with conventional and proposed Doppler measurements is assessed.

Here tight integration uses only Doppler measurements to update the user velocity and heading. No pseudorange measurements are used. In this way, the accumulating errors caused by inaccurate Doppler measurements will become more apparent. The other reason to choose HSGPS/PDR integration with conventional and proposed Doppler measurements is to make the results comparable. If the velocity MLE is directly integrated with PDR, it is then a loose integration scheme. This might obscure the benefits of the measurements and integration schemes. By using the same integration scheme, the benefits of better Doppler measurements will become evident.

For the DR algorithms, the step event is first detected by using pattern recognition techniques with a MEMS accelerometer [24] and the step length is calculated by using an averaged reference velocity. In this way, the step length estimation error will be very small. The methods of step length estimation have reached a good and consistent level of accuracy, especially with foot and belt mounted sensors, thus it is not a concern in this paper. Only the long term heading accuracy is the focus of the paper. Regarding the PDR heading information, only one tactical grade vertical gyroscope rigidly attached to the backpack is used to provide heading angles with accumulated errors. And such heading angles are fed directly to the PDR filter.

**Figure 10.**
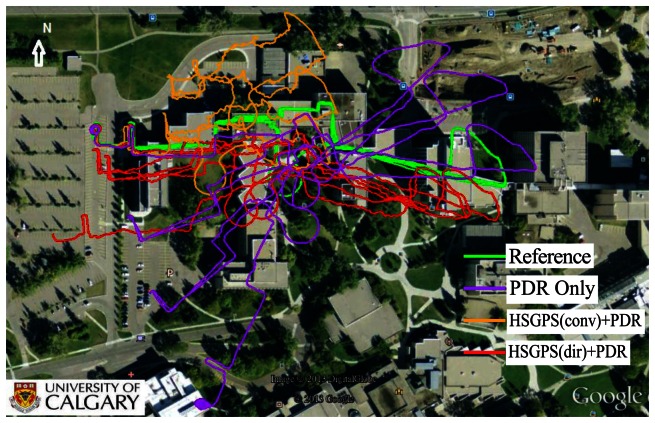
Trajectories of various navigation solutions.

The trajectories of PDR only and HSGPS/PDR tight integration with conventional and proposed Doppler measurements are shown in Figure 10. It can be seen that the PDR only solution is much smoother, which shows better short-term accuracy. However, as time evolves, the PDR only solution is rotated due to the large heading drift and is biased by hundreds of metres. When integrating PDR with conventional HSGPS, the shape of the trajectory is severely distorted. On the other hand, integrating PDR with the proposed approach gives a solution which alleviates the heading drift and better preserves the shape of the trajectory as compared to the other two cases.

**Figure 11.**
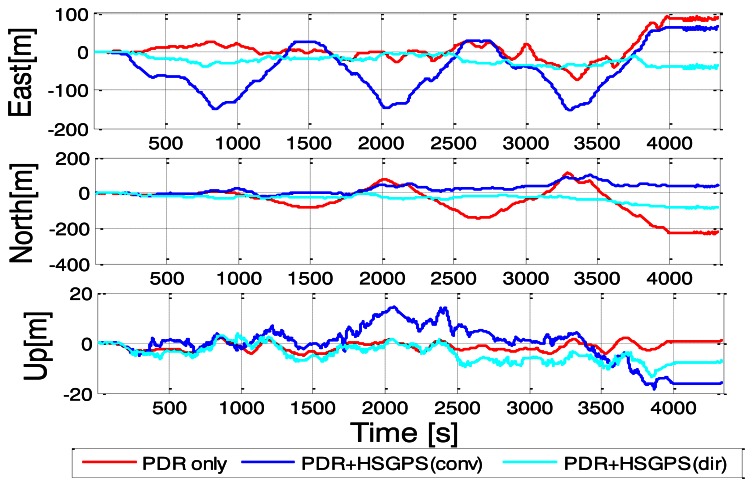
Position errors of various navigation solutions.

**Figure 12.**
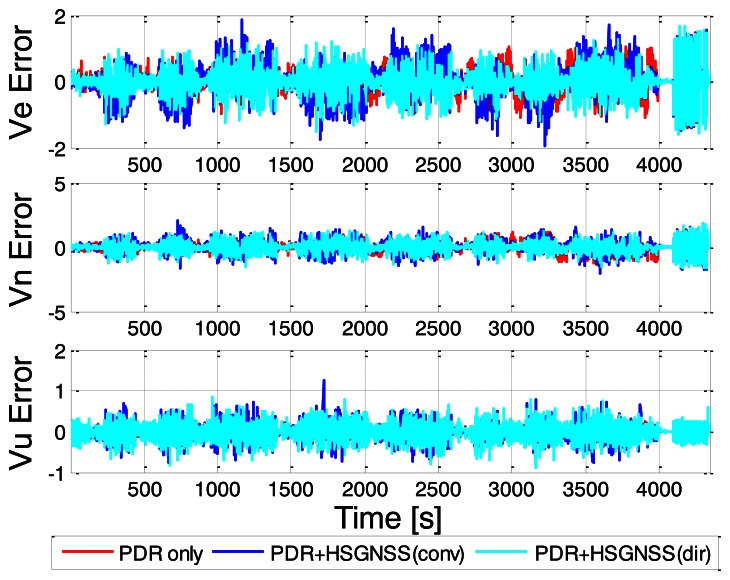
Velocity errors of various navigation solutions.

Among all three navigation solutions, it can be seen that the navigation solution with the proposed Doppler measurements integrated with PDR is the nearest to the reference trajectory, which validates the effectiveness of the proposed method. In order to further show the error characteristics, the position and velocity errors are plotted as a function of time in Figures 11 and 12.

**Figure 13.**
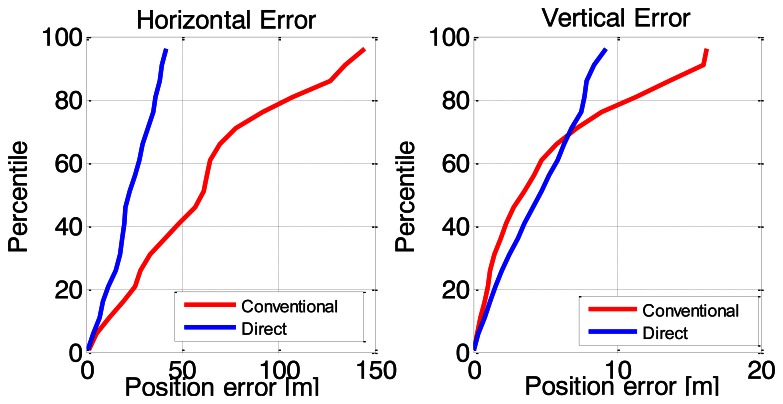
Cumulative histograms of horizontal and vertical position errors.

To further analyze the results, the cumulative histograms of the horizontal and vertical position errors are given in Figure 13. Two key things are worth noting. First, the errors are clearly non-Gaussian, which is not surprising given the operating environment and the high probability of highly-varying NLOS errors. Second, it can be observed that the proposed method has considerably better horizontal positioning performance compared to the conventional HSGPS/PDR solution. For the vertical position error, both solutions are similar until about 7 m, at which point the proposed method begins to outperform the conventional HSGPS/PDR solution.

The corresponding RMS error statistics are summarized in Table 3. As shown, the east position errors of conventional HSGPS/PDR solution are nearly twice that of the PDR-only solution. Regarding the north position RMS errors, it can be seen that the improvement of the proposed solution over conventional approach is not as apparent as east direction. This is very likely due to the fact that the user displacements along the east-west axis are much larger than the displacements along north-south direction for the whole trajectory. Thus the position errors in north direction are not as easily observed as east direction. Similarly, for the east velocity errors, it is observed that the conventional HSGPS/PDR integrated solution also degrades performance. This is because the Doppler MLEs in the indoors are not as reliable and may even bias the results. However, the direct vector HSGPS/PDR integrated navigation solution shows that both the position and the velocity RMS errors have noticeable improvements as compared to PDR-only solution. For example, the east, north and RMS position errors of the proposed algorithm have improvements of 24% and 60% as compared to PDR-only solution. Similarly, the north velocity accuracy has an improvement of 13% as compared to PDR-only solution. One can also observe that there is 0.03 m/s degradation in the east velocity when integrating direct vector HSGPS with PDR. However, the direct vector HSGPS/PDR velocity still outperforms conventional HSGPS/PDR velocity by 16%, and 9% in the east and north axes, respectively.

These results suggest that direct vector HSGPS Doppler actually reduces such errors on average, which, as discussed below, improves heading determination. For this data set, the benefit from direct vector HSGPS Doppler is more significant in the north direction. To further illustrate the improvement, the mean velocity errors are shown in Table 4. It can be seen that the proposed method generally outperforms the PDR only solution in both the east and north axis, and the biasness of east velocity has a slight improvement with respect to conventional approach.

**Figure 14.**
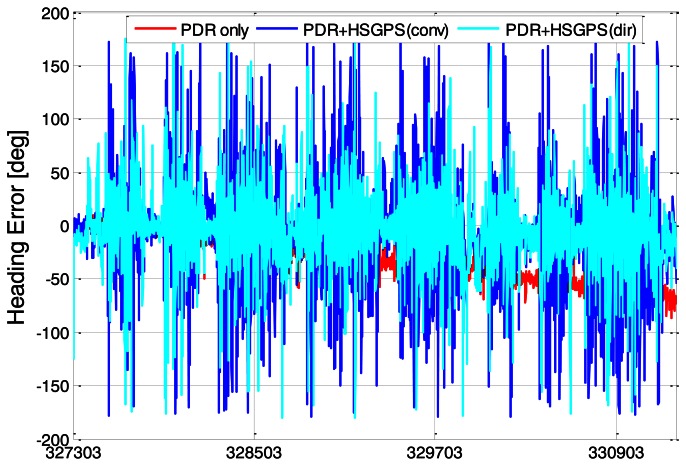
Interpolated heading errors.

In Figure 14, the estimated headings are plotted. It can be seen that the error of the proposed method is generally smaller than with conventional HSGPS/PDR and does not exhibit the long-term drift seen in the PDR-only heading.

## 5. Conclusions

In this paper, the combined indoor signal and multipath model is first introduced. How multipath affects conventional HSGNSS Doppler measurements is then discussed. After that direct vector receiver architecture is proposed and compared to conventional high sensitivity GNSS receivers. The conventional method performs well in most good scenarios, and the proposed method is the maximum likelihood extension to the conventional method, which will asymptotically approach the performance bound. How velocity and Doppler measurements are obtained with direct vector processing performing in indoor multipath environments is also investigated. In order to evaluate the benefits of Doppler estimated with the proposed direct vector processing method over conventional block processing method, Doppler estimated with both approaches are tightly integrated in a detailed navigation filter, which follows a PDR strategy. Comparisons are made between PDR only solution and integrated solutions. From the experimental results, the following conclusions can be drawn:

- In indoor environments, the direct vector processing makes the best use of LOS and NLOS signal power for each satellite. Additionally, the mutual information between satellites is further used before arriving at a solution.
- Velocity estimation by using direct vector HSPGS outperforms the conventional HSGPS for two indoor scenarios considered herein. The HSGPS Doppler measurements obtained by using direct vector processing are thus more reliable and helpful than conventional HSGPS Doppler measurements for heading estimation.
- In weak signal and multipath conditions, the benefit of using conventional HSGPS Doppler measurements is very limited due to the fact that proper weighting cannot be correctly set. However, the proposed direct vector processing method autonomously weighs the navigation solution according to power (equivalent to C/N_0_), which is actually the maximum likelihood estimate of the navigation solution, and performs no worse or better than the conventional approach.
- By integrating the Doppler measurements from the proposed direct vector processing with PDR, the solutions show improvements in east and north velocity estimation of 16% and 9% for the indoor scenarios considered herein as compared to the conventional approach.

Currently only the GPS system in L1 band is used. In an on-going project, the GLONASS signals in L1 and L2 bands will also be included. It can be expected that with increased number of satellites, the proposed Doppler will have a tendency to be closer to the true value under certain circumstances. Various indoor environments will be assessed in order to show the strength and weakness of the methods proposed in this paper. Regarding the integration, other heading sensors, such as magnetometers, and low-cost MEMS gyroscopes will also be included, and the benefits of using HSGNSS Doppler measurements in various indoor environments will be further assessed and analyzed.

## Figures and Tables

**Table 1.** Velocity RMS errors–indoor scenario A.

**Velocity RMS Errors**	**Direct**	**Conventional**	**Improvement**
East (m/s)	0.14	0.56	75%
North (m/s)	0.32	1.29	75%
Up (m/s)	0.52	4.96	89%

**Table 2.** Velocity RMS errors–indoor scenario B.

**Velocity RMS Errors**	**Direct**	**Conventional**	**Improvement**
East (m/s)	0.19	0.25	24%
North (m/s)	0.32	1.16	72%
Up (m/s)	0.41	2.43	83%

**Table 3.** Position and velocity RMS errors.

	**Position RMS Errors (m)**			**Velocity RMS Errors (m/s)**		
	
	**East**	**North**	**Up**	**East**	**North**	**Up**
PDR-only	33.65	93.18	2.23	0.38	0.47	0.16
PDR+HSGPS (conv.)	75.71	35.41	7.55	0.49	0.45	0.20
PDR+HSGPS (dir.)	25.52	37.45	5.53	0.41	0.41	0.20

**Table 4.** Mean velocity errors.

	**Velocity Mean errors (m/s)**		

	**East**	**North**	**Up**
PDR-only	0.02	−0.06	0.00
PDR+HSGPS (conventional)	0.02	0.01	0.00
PDR+HSGPS (direct)	−0.01	−0.01	0.00
